# Genome-wide association study of toxic metals and trace elements reveals novel associations

**DOI:** 10.1093/hmg/ddv190

**Published:** 2015-05-29

**Authors:** Esther Ng, P. Monica Lind, Cecilia Lindgren, Erik Ingelsson, Anubha Mahajan, Andrew Morris, Lars Lind

**Affiliations:** 1 Wellcome Trust Centre for Human Genetics, Oxford OX3 7BN, UK,; 2 Department of Medical Sciences, Occupational and Environmental Medicine, Uppsala University, Uppsala SE-751 85, Sweden,; 3 Department of Medical Sciences, Molecular Epidemiology and Science for Life Laboratory,; 4 Department of Medical Sciences, Cardiovascular Epidemiology, Uppsala University, Uppsala, Sweden,; 5 Broad Institute of the Massachusetts Institute of Technology, Harvard University, Cambridge, MA 02142, USA and; 6 Department of Biostatistics, University of Liverpool, Liverpool L69 3BX, UK

## Abstract

The accumulation of toxic metals in the human body is influenced by exposure and mechanisms involved in metabolism, some of which may be under genetic control. This is the first genome-wide association study to investigate variants associated with whole blood levels of a range of toxic metals. Eleven toxic metals and trace elements (aluminium, cadmium, cobalt, copper, chromium, mercury, manganese, molybdenum, nickel, lead and zinc) were assayed in a cohort of 949 individuals using mass spectrometry. DNA samples were genotyped on the Infinium Omni Express bead microarray and imputed up to reference panels from the 1000 Genomes Project. Analyses revealed two regions associated with manganese level at genome-wide significance, mapping to 4q24 and 1q41. The lead single nucleotide polymorphism (SNP) in the 4q24 locus was rs13107325 (*P*-value = 5.1 × 10^−11^, *β* = −0.77), located in an exon of *SLC39A8*, which encodes a protein involved in manganese and zinc transport. The lead SNP in the 1q41 locus is rs1776029 (*P*-value = 2.2 × 10^−14^, *β* = −0.46). The SNP lies within the intronic region of *SLC30A10*, another transporter protein. Among other metals, the loci 6q14.1 and 3q26.32 were associated with cadmium and mercury levels (*P* = 1.4 × 10^−10^, *β* = −1.2 and *P* = 1.8 × 10^−9^, *β* = −1.8, respectively). Whole blood measurements of toxic metals are associated with genetic variants in metal transporter genes and others. This is relevant in inferring metabolic pathways of metals and identifying subsets of individuals who may be more susceptible to metal toxicity.

## Introduction

Toxic metals are ubiquitous in the environment, with sources ranging from shipyard and construction business to agricultural and domestic industries. These metals are bio-accumulative and have a variety of toxic effects in the human body (1). For example, exposure to cadmium could result in liver and renal damage, as well as bone demineralization, while lead poisoning could affect the gastrointestinal and neurological systems (2).

The accumulation of these metals in the body is not only governed by environmental exposure but also by mechanisms involved in the absorption, distribution, metabolism and elimination (ADME) of the compounds. Knowledge about the pathways involved in the ADME of different metals has generally been generated in experimental studies.

For example, several gene transporter families have been implicated in manganese transport in plants (3). These transporter families include *NRAMP* (natural resistance-associated macrophage protein), *YSL* (yellow stripe-like), *ZIP* [zinc-regulated transporter/iron-regulated transporter (*ZRT/IRT1*)-related protein], *CAX* (cation exchanger), *CCX* (calcium cation exchangers), *CDF/MTP* (cation diffusion facilitator/metal tolerance protein) and *VIT* (vacuolar iron transporter).

Another example is that the P(1B)-type heavy metal ATPases (HMAs) are diverse in terms of tissue distribution, subcellular localization and metal specificity. Functional studies of HMAs have shown that these transporters can be divided into two subgroups based on their metal–substrate specificity: a copper (Cu)/silver (Ag) group and a zinc (Zn)/cobalt (Co)/cadmium (Cd)/lead (Pb) group (4).

However, it is not known if all of these mechanisms involved in the ADME of metals are operating and valid in humans. One way to examine such mechanisms in humans is to evaluate if genetic variation in the genes encoding the critical proteins involved in these ADME steps are related to circulating levels of relevant metals. Mendelian disorders such as Wilson's disease and Menke's disease are examples of direct genetic influence on metal ADME steps. The former is an autosomal recessive disorder involving a mutation in ATP7B gene, causing abnormal hepatic copper deposition, and the latter is an X-linked recessive disorder involving a mutation in the ATP7A gene, causing copper deficiency. Additionally, hereditary factors and environmental factors together have been suggested to influence blood levels of cadmium and lead in healthy individuals (5). A twin study using genetic linkage analysis showed significant linkage for lead and suggestive linkage for cadmium, mercury, selenium and zinc (6).

Today, we have the ability to investigate genetic variation at multiple sites across all chromosomes through genome-wide association studies (GWAS). This approach has the advantage that associations with genetic variation mapping to any gene, not just some pre-specified subset, can be investigated. So far, there has been one GWAS that examined the levels of copper, zinc and selenium, revealing several loci with biologically relevant genes (7). For example, the locus showing association with zinc contains genes for the Zn-containing enzyme carbonic anhydrase, and the locus associated with selenium includes genes involved in the metabolism of sulphur-containing amino acids. Identification of these loci may help to identify population subgroups that may be more susceptible to deficiency or toxicity.

Using a GWAS approach, our study aimed to investigate genetic variants associated with serum levels of a range of metals in a cohort of 949 individuals. The metals were aluminium, cadmium, cobalt, copper, chromium, mercury, manganese, molybdenum, nickel, lead and zinc. To our knowledge, this is the first GWAS studying serum levels of toxic metals, in a comprehensive manner and not restricted to trace elements.

## Results

A total of 949 individuals from the Prospective Investigation of the Vasculature in Uppsala Seniors (PIVUS) study were included in the sample. Following imputation, a total of 8 736 858 high-quality single nucleotide polymorphisms (SNPs) with minor allele frequency (MAF) > 1% were included in the analysis. Median values for the studied metals are presented in Table 1, together with the genomic control inflation factor, all of which were close to 1. No additional correction for residual population structure after adjustment for principal components was required. Further details are available in Materials and Methods.

**Table 1.** Median values (with 25th and 75th percentiles) and genomic control lambda are given for the different metals included in the study

Metal	Median serum level (SD) (mmol/l)	Genomic inflation factor
Al	0.637 (0.504–0.795)	1.02
Cd	2.41 (1.72–3.74)	1.00
Co	1.43 (1.09–1.90)	1.00
Cr	11.8 (9.58–16.00)	1.01
Cu	12.8 (11.6–14.1)	1.00
Hg	8.92 (5.86–13.65)	1.00
Mn	138 (115–165)	1.01
Mo	9.74 (8.09–11.80)	0.991
Ni	90.6 (68.3–109.0)	0.982
Pb	0.083 (0.059–0.110)	1.01
Zn	96.0 (87.7–104.0)	1.02

A total of three metals demonstrated signals at a genome-wide significance level—manganese, cadmium and mercury—defined here as *P* < 4.5 × 10^−9^ with a Bonferroni correction for 11 tested metals.

**Figure 1.**
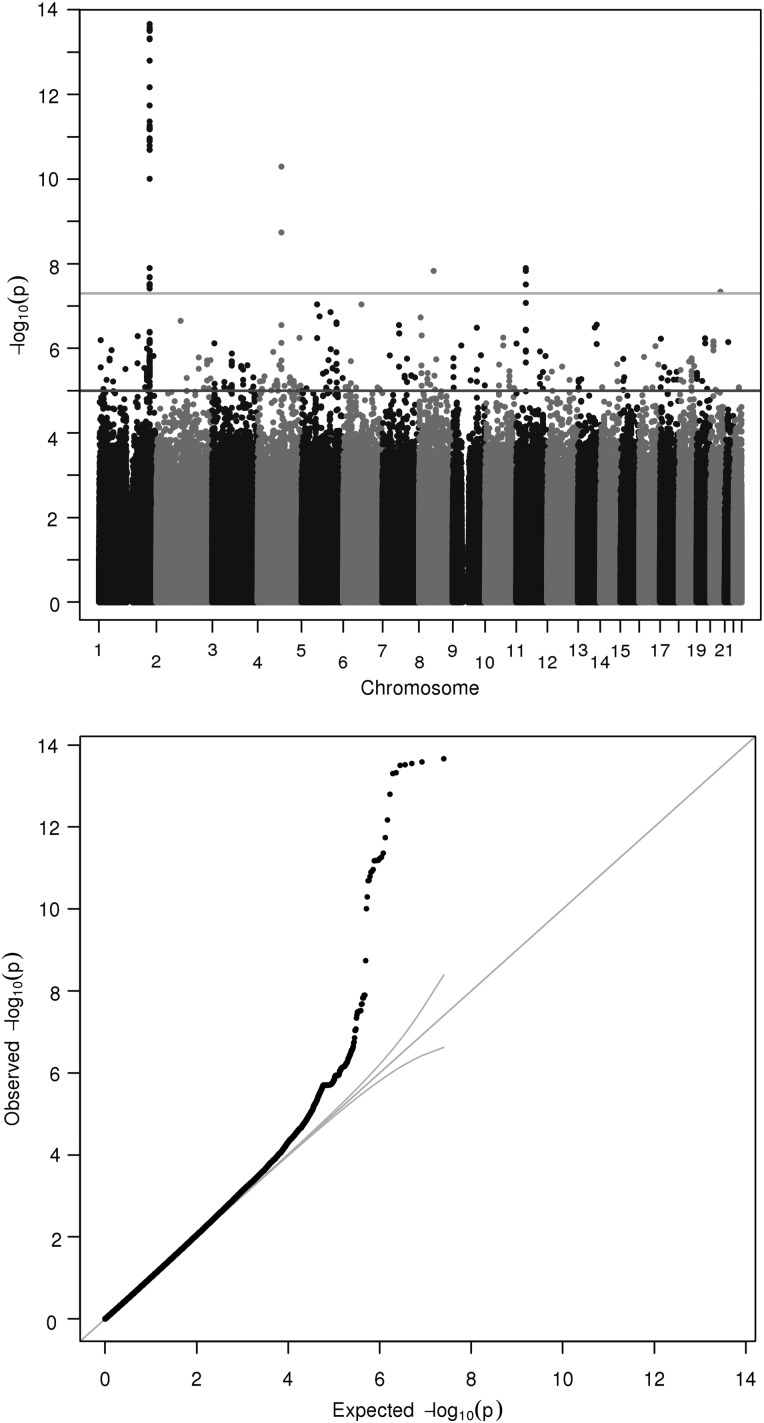
Manhattan and quantile–quantile plot of GWAS of serum manganese. Each point corresponds to a SNP passing quality control (QC), plotted according to genomic position on the *x*-axis and the strength of association (−log_10_ *P*-value) on the *y*-axis. The horizontal lines indicate genome-wide significance threshold (5 × 10^−8^) and also a nominal threshold of 10^−05^.

The GWAS analyses revealed two regions that were associated with manganese level. These mapped to 4q24 and 1q41 (Fig. 1 shows the Manhattan and quantile–quantile plots). The lead SNP in the 4q24 locus was a coding variant, rs13107325 (*P*-value of 5.08 × 10^−11^, *β* value of −0.767, MAF of 0.038), located in *SLC39A8*, a gene that encodes a well-known protein involved in manganese and zinc transport (8). The amino acid change for this SNP was alanine to threonine. ‘Sorting Tolerant From Intolerant’ (SIFT) algorithm (9) prediction revealed that the variation may have damaging effects on protein formation. The lead SNP in the 1q41 locus was rs1776029 (*P*-value of 2.17 × 10^−14^, *β* value of −0.456, MAF of 0.182). The SNP lies just downstream of *SLC30A10*, another well-established ion transporter protein (10). There are no non-synonymous SNPs in the region that pass the genome-wide significance threshold, and conditional analysis revealed no secondary signals. Supplementary Material, Figs S1 and S2 show the local association plots for these two loci. The lead SNP rs1776029 lies within a region known to be marked by histone-3-lysine-27 trimethylation (H3K27Me3), which is thought to be inhibitory to transcription (11).

**Figure 2.**
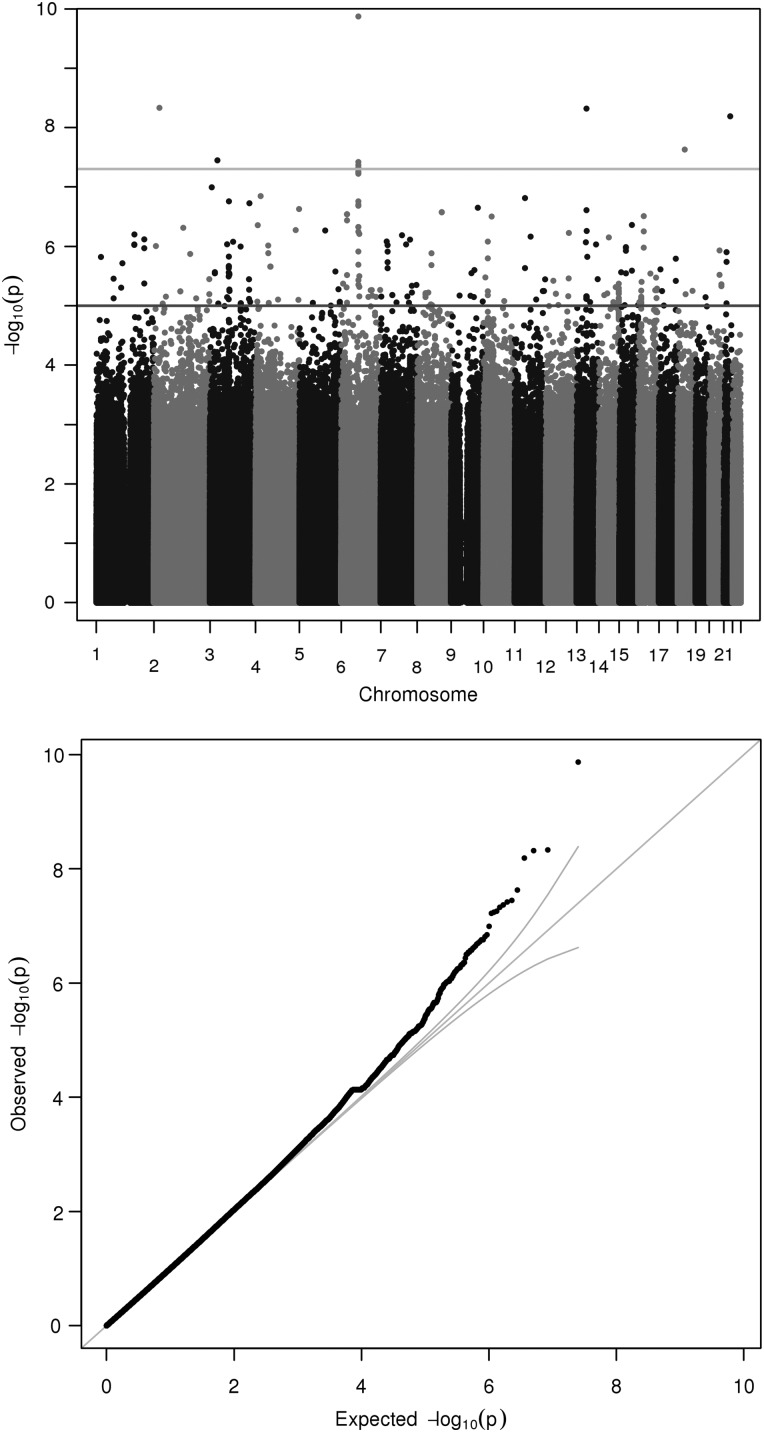
Manhattan and quantile–quantile plot of GWAS of serum cadmium. Each point corresponds to a SNP passing QC, plotted according to genomic position on the *x*-axis and the strength of association (−log_10_ *P*-value) on the *y*-axis. The horizontal lines indicate genome-wide significance threshold (5 × 10^−8^) and also a nominal threshold of 10^−05^.

The locus 6q14.1 was associated with cadmium levels. The lead SNP was rs9350504 (*P*-value of 1.35 × 10^−10^, *β* value of −1.16, MAF of 0.0175), which is an intronic variant within CD109, a glycosyl phosphatidylinositol-linked glycoprotein (Fig. 2). Since smoking was correlated with cadmium levels, we also tested the model with smoking included as a covariate. In this model, the *P*-value of rs9350504 increased marginally to 1.90 × 10^−11^. This SNP lies within a region known to be marked by H3K27Me3 as well as histone-3-lysine-4 monomethylation (H3K4Me1), the latter of which is thought to be an enhancer of transcription (12).

**Figure 3.**
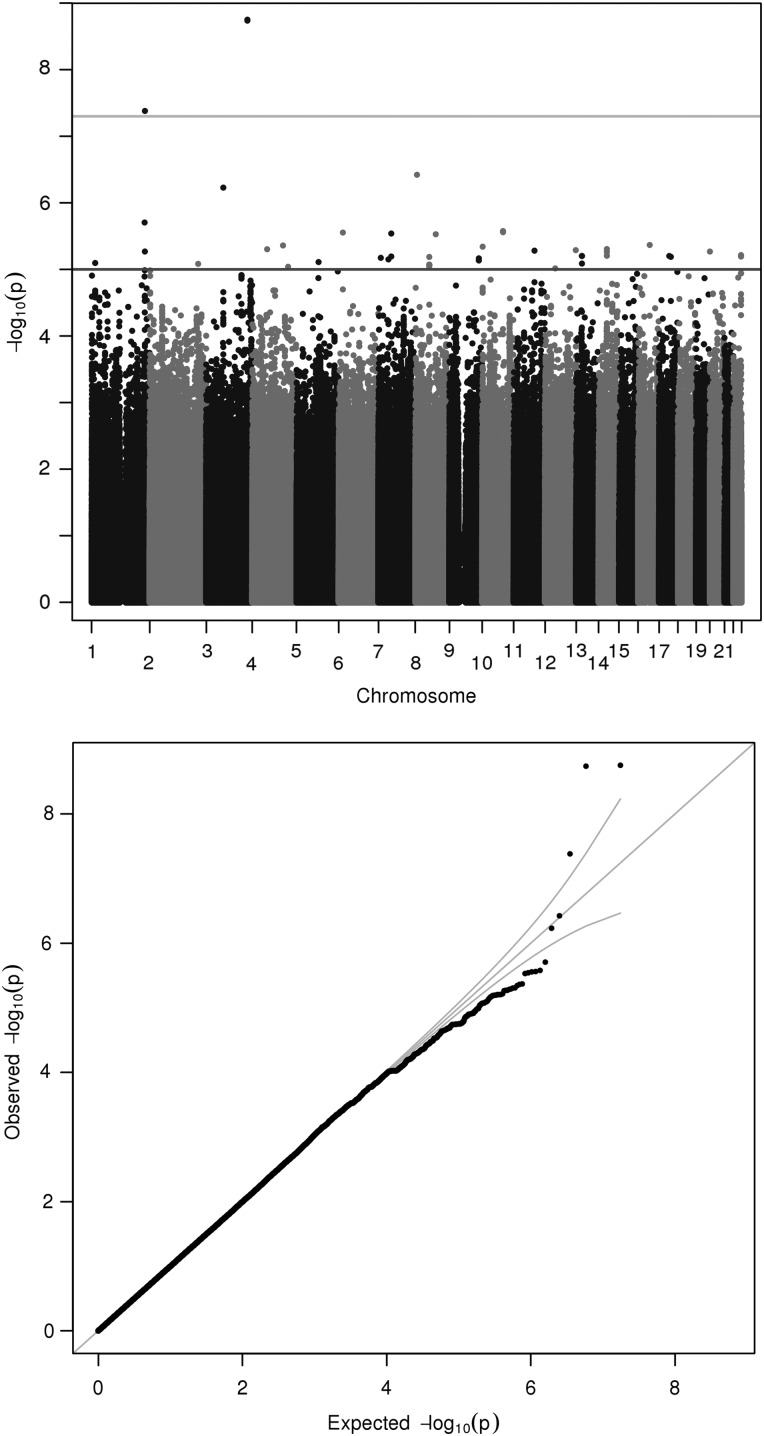
Manhattan and quantile–quantile plot of GWAS of serum mercury. Each point corresponds to a SNP passing QC, plotted according to genomic position on the *x*-axis and the strength of association (−log_10_ *P*-value) on the *y*-axis. The horizontal lines indicate genome-wide significance threshold (5 × 10^−8^) and also a nominal threshold of 10^−05^.

The 3q26.32 locus was associated with mercury levels (*P*-value of 1.76 × 10^−9^, *β* value of −1.80, MAF of 0.0140). The lead SNP is rs148534631, an intron variant within *LINC00578*, an intergenic non-protein-coding RNA (Fig. 3). Local association plots for these two metals can be found in Supplementary Material, Figs S2 and S3. The lead SNP rs148534631 also lies within a region known to be marked by H3K27Me3.

Supplementary Material, Table S2 provides a list of lead SNPs associated with metals at a *P*-value threshold of 1 × 10^−5^. A SNP associated with chromium levels (rs12915189) was associated with information processing speed in a GWAS on three cohorts of individuals (13). This SNP is an intron variant in *CRTC3*, which regulates CREB-dependent gene transcription in a phosphorylation-independent manner. A SNP associated with manganese (rs11006464) was associated with oleic acid levels in a GWAS on five population-based cohorts (14). It is an intron variant in the *FAM13C1* gene.

When comparing our results with that of Evans *et al.* (7), the SNPs that showed genome-wide significance in their study for Zn levels (rs1532423 and rs2120019) had *P*-values of 0.000142 and 1.03 × 10^−5^ in our study and same direction of effects, demonstrating consistency with other cohorts.

## Discussion

The present study showed that the GWAS approach is useful to investigate mechanisms in humans that are of interest for circulating levels of toxic metals and trace elements. By this approach, we identified novel associations between genetic variation mapping to genes encoding for proteins known to be involved in ion transport in experimental studies. This approach could validate that these previously described ADME pathways are also valid in humans. Besides the associations with ion transporters, we also found an association between cadmium and *CD109* not previously described. As evidenced by the negative *β* values, for all four significant SNPs, the presence of minor alleles is associated with lower metal serum levels compared with the median values, possibly suggesting increased clearance or decreased absorption.

Manganese is a trace metal that is essential to the body, but can also be neurotoxic at higher chronic exposure levels, with symptoms persisting long after cessation of exposure. Occupational studies have demonstrated that exposure to manganese can cause cognitive and motor defects, in addition to changes in mood and short-term memory, altered reaction time and reduced hand–eye coordination (15,16). Affected workers often show accumulation of manganese in the globus pallidus (17). Serum manganese levels can be influenced by a range of factors, including dietary characteristics for example carbohydrate source, presence of phytate and presence of animal protein and dietary iron (18). In addition, serum ferritin concentration has also been associated with serum manganese levels (19,20). Henn *et al.* studied a cohort of 332 women and reported that women with human haemachromatosis protein (*HFE*) variant alleles at rs1800562 or rs1799945 had 12% lower blood manganese concentrations than women with no variant alleles. The reason for this is postulated to be a common cellular transport mechanism; hence, excess of one element inhibits transport of the other (21). In our study, rs1800562 was weakly associated with manganese levels (*P*-value of 0.000484) but not rs1799945 (*P*-value of 0.817).

In our study, rs13107325 was associated with manganese levels. Several studies have uncovered an association between this SNP and other phenotypes, including metabolic traits such as blood pressure, body mass index and lipid levels (22–25). Rs13107325 is a non-synonymous SNP within SLC39A8. This gene encodes a zinc transporter that functions in the cellular importation of zinc at the onset of inflammation. Waterworth's reports postulate that since the gene is induced by tumour necrosis factor alpha (TNF-α), it is possible that the SLC39A8 molecule might be associated with lipids such as high-density lipoprotein (HDL) in an inflammatory context. This SNP has also been associated with neurological phenotypes including schizophrenia (26) and stress (27).

This SNP gives rise to substitution of alanine (hydrophobic) to alanine (hydrophilic). It is in low linkage disequilibrium (LD) with nearby SNPs in HapMap sample (26). *SLC39A8* is one of the members of the family of 39 solute carrier transporters. It transports manganese and zinc, which are crucial for the functioning of various organs, but may be neurotoxic at levels beyond a certain concentration. Excessive exposure to manganese may lead to Parkinsonian features, as well as other neuropsychiatric symptoms such as agitation and hallucination. *SLC39A8* is responsible for the uptake of manganese and zinc from blood into various tissues. It is expressed at high levels in various tissues (including placenta), but low levels in the brain. The lead SNP in the 1q41 locus is rs1776029. The SNP lies within the intronic region of *SLC30A10*, which is a member of the solute carrier family 30 member 10. *SLC30A10* is a member of the *SLC30* solute carrier subfamily of the cation diffusion facilitator (28). It is a manganese efflux transporter localized to the cell surface, which decreases intracellular manganese and protects against manganese toxicity (29). Recessive mutations in SLC30A10 have been linked to a syndrome characterized by severe manganese accumulation, polycythaemia, neurological disturbances and liver complications (10,30). Of note, these patients were not known to be exposed to excessive manganese, yet they developed symptoms of toxicity. *SLC30A10* is the only mutated protein known to cause manganese toxicity, suggesting that it may play a key role in manganese clearance (31). Of note, it was originally suggested to be a zinc transporter; however, in our study, serum zinc levels were not significantly associated with the presence of mutation (28).

Cadmium is a common environmental pollutant, with exposure arising from tobacco smoke (32), foods such as shellfish, liver and kidney, as well as inhalation of cadmium-contaminated air from industry sectors. Adverse health effects have been reported in association with low-level environmental cadmium exposure, including liver and kidney damage (33). In addition, there is increased risk of cancer, including breast, kidney, pancreas and urinary bladder (34).

The genetic basis of cadmium ADME is poorly understood. A SNP in the core promoter region of metallothionein 2A (*MT2A*), rs28366003, has previously been shown to be associated with cadmium levels (*P*-value = 0.004 in the present sample) (35). Metallothioneins (MTs) are low-molecular weight, cysteine-rich proteins that bind to the biologically essential metals and perform these metals' homeostatic regulations; absorb the heavy metals and assist with their transportation and extraction. In another study on 172 Andean women, a polymorphism in the transferrin receptor gene *TFRC*, rs3804141, was associated with urinary cadmium levels, with mean urinary cadmium concentrations that were 22% higher in women with GA and AA genotypes (*P*-value = 0.78 in the present sample when related to circulating Cd concentrations) (36). Our study demonstrated an association between cadmium blood levels and an SNP within the *CD109* gene. *CD109* encodes a glycosyl phosphatidylinositol-linked glycoprotein that localizes to the surfaces of endothelial cells, T cells and platelets. It is associated with various cancers. For example, upregulation of *CD109* is associated with oral cancer and lung cancer (37,38). High expression of *CD109* antigen is associated with poor prognosis of soft tissue sarcoma (39), and *CD109*+ endothelial cells is a prognostic indicator for glioblastomas (40). It is of interest to note that cadmium exposure also has been discussed in the aetiology of several cancers (41,42). Nevertheless, *CD109* now emerges as a new player in cadmium ADME, but this has to be replicated in an independent sample.

A few genetic variants have previously been associated with mercury toxicokinetics, mainly in the glutathione-related genes (33,43,44), as well as multi-specific transporter family genes (45). However, there have been no genome-wide studies that address this question. Our study demonstrates an association between mercury levels and a SNP in *LINC00578*, but the function of this intergenic non-protein-coding RNA is not clear.

The overlap between these significant SNPs and regions of known histone modification is notable since the latter may enhance or repress nearby gene transcription. The fact that the top SNP for cadmium (rs9350504) lies within the region known to be marked by both H3K27Me3 and H3K4Me1 is particularly interesting because the former is a repressor of transcription while the latter is thought to be an enhancer (12). In the genome, there are regions hypothesized to exist in a ‘poised’ state. These bivalent domains tend to coincide with transcription factor genes expressed at low levels and have been postulated to silence developmental genes in embryonic stem cells while keeping them in a state ready for activation (46).

This study includes a large number of metals and trace elements measured in sample for which extensive genotyping and imputation have been performed. However, statistical power is limited due to correction for multiple testing; hence, only large gene versus phenotype effects could be discovered. Also, the GWAS-significant findings in the present study have to be replicated in order to be fully validated. The SNPs found to be associated with metal levels in this study may not be the biological cause of association but rather in LD with causative variants; hence, it may be interesting to further investigate this possibility through deep sequencing in associated LD blocks.

A GWAS approach to study association between genetic variation and circulating levels of toxic metals and some trace elements disclosed novel associations between some ion transporters and whole blood manganese concentrations. In addition, other less well-known genes were related to cadmium and mercury concentrations, showing GWAS to be a valuable tool to explore ADME pathways for metals in humans.

## Materials and Methods

The PIVUS study was initiated in 2001 to investigate the predictive power of different measurements of vascular characteristics for future cardiovascular events. Secondary aims included measurements of cardiac and metabolic functions, as well as serum biomarkers and levels of environmental pollutants including polychlorinated biphenyls. Sample details and methods are described more fully by Lind *et al*. (47). Briefly, subjects aged 70 years living in the community of Uppsala were randomly selected from the community register and invited to participate in the study. Of the 2025 subjects invited, 1016 subjects agreed to participate. After excluding individuals for which phenotypical data were missing, 949 subjects remained.

All 11 toxic metals and trace elements (Al, Cd, Co, Cu, Cr, Hg, Mn, Mo, Ni, Pb and Zn) in this study were determined in whole blood. The analysis was performed using inductively coupled plasma sector field mass spectrometry, after microwave-assisted digestion with nitric acid (48). Details on the analysis have been described by Lind *et al*. (49).

DNA samples were genotyped according to the manufacturer's instructions on Illumina Infinium Omni Express bead microarrays. Heterozygosity was calculated as the proportion of autosomal SNPs at which the individual carries a heterozygous genotype. Samples were excluded if the call rate was <95%, if they had extreme heterozygosity (>3 SD from the mean), if they were ethnic outliers, or if they were gender discordant. SNP quality control measures included exact *P*-value for deviation from Hardy–Weinberg equilibrium <10^−10^ and missing genotype rate >0.01 (MAF < 5%) or missing genotype rate >0.05 (MAF ≥ 5%). Multidimensional scaling was performed to obtain principal components to adjust for population structure. Prior to imputation, variants with MAF < 1% were removed from the GWAS scaffold. Samples were pre-phased with SHAPEIT2. Genotype data were then imputed up to the ‘all ancestries’ reference panel from the 1000 Genomes Project Consortium Phase 1 interim release (June 2010) (50) using IMPUTEv2 (51,52).

Metal measurements were then ranked inverse normalized to generate a Gaussian distribution for downstream association analyses and minimize the impact of outliers. Association testing for each transformed metal was performed in a linear regression framework under an additive model in the minor allele for each variant in turn, after adjusting for triglycerides, cholesterol, gender and two principle components from multidimensional scaling to account for population structure as covariates. Age was not included as a covariate because all individuals were of the same age. Association testing was performed in SNPTEST, allowing for uncertainty in the imputation in a missing data likelihood. The association analysis was restricted to SNPs with MAF > 1% and imputation quality information score (info) of >0.4. The genomic control inflation factor (52,53) for each metal was used to assess evidence of residual population structure. Conditional analyses to search for secondary signals of association were performed by including genotype dosage at the lead SNP as an additional covariate in the linear regression model. To assign function to these genomic variants, we annotated all SNPs that met a significance criterion of 10^−5^ using ANNOVAR (53) and the Encyclopaedia of DNA variants (ENCODE *et al*.) (54).

## Supplementary Material

Supplementary Material is available at *HMG* online.

## Funding

Financial support was received from the Wellcome Trust (grant numbers WT098017, WT090532, 097306/Z/11/Z and WT064890). C.M.L. is a Wellcome Trust Research Career Development Fellow (086596/Z/08/Z). Funding to pay the Open Access publication charges for this article was provided by Wellcome Trust.

## Supplementary Material

Supplementary Data
