# Supplementary material for: Genome-wide association study of toxic metals and trace elements reveals novel associations
Source: Hum Mol Genet. 2015 May 29;24(16):4739–45. doi: 10.1093/hmg/ddv190 (PMC4512629; doi:10.1093/hmg/ddv190)
Supplement: Supplementary Data [file supp_24_16_4739__index.html]

Genome-wide association study of toxic metals and trace elements reveals novel associations — Genome-wide association study of toxic metals and trace elements reveals novel associations — Supplementary Data 

# Genome-wide association study of toxic metals and trace elements reveals novel associations

## Supplementary Data

Supplementary Data

- Supplementary Data - Docx file
