# Supplementary material for: Genome-wide association study of toxic metals and trace elements reveals novel associations
Source: Hum Mol Genet. 2015 May 29;24(16):4739–45. doi: 10.1093/hmg/ddv190 (PMC4512629; doi:10.1093/hmg/ddv190)

SUPPLEMENTARY MATERIAL

Table 1 Lead SNPs which are associated with metals at p value less than 10^-5^

| Pollutant | Chr | coordinate | | Allele 1 | | Allele 2 | | MAF | p value | Beta | Standard Error | Location | Nearest_Gene |
| --- | --- | --- | --- | --- | --- | --- | --- | --- | --- | --- | --- | --- | --- |
| Al | 8 | 9095620 | | C | | T | | 0.0106 | 2.63E-07 | 1.50 | 0.291 | intergenic | PPP1R3B(dist=86468),RP11-115J16.1(dist=86941) |
| Al | 2 | 205169549 | | C | | A | | 0.0384 | 3.12E-07 | 0.755 | 0.148 | intergenic | ICOS(dist=343251),PARD3B(dist=240967) |
| Al | 2 | 108883300 | | T | | C | | 0.0142 | 4.81E-07 | 1.47 | 0.293 | intergenic | SULT1C3(dist=1493),SULT1C2(dist=21795) |
| Al | 13 | 85406657 | | C | | T | | 0.0332 | 1.57E-06 | -0.686 | 0.143 | intergenic | LINC00333(dist=225754),LINC00351(dist=531081) |
| Al | 1 | 206986926 | | C | | T | | 0.491 | 1.92E-06 | 0.223 | 0.0469 | intronic | IL19 |
| Al | 13 | 51129735 | | A | | C | | 0.0171 | 3.72E-06 | 1.10 | 0.238 | intergenic | DLEU1(dist=26956),DLEU7(dist=157024) |
| Al | 2 | 138130617 | | A | | T | | 0.0198 | 3.78E-06 | 1.09 | 0.235 | intronic | THSD7B |
| Al | 9 | 115848169 | | T | | A | | 0.170 | 5.95E-06 | 0.316 | 0.0698 | intergenic | ZFP37(dist=29098),FAM225B(dist=18834) |
| Al | 15 | 64992602 | | A | | T | | 0.0151 | 6.03E-06 | 1.27 | 0.280 | intronic | OAZ2 |
| Al | 15 | 99927371 | | T | | C | | 0.0911 | 6.43E-06 | 0.484 | 0.107 | downstream | LRRC28 |
| Al | 7 | 89168136 | | C | | T | | 0.0969 | 7.05E-06 | 0.373 | 0.0830 | intergenic | ZNF804B(dist=201790),STEAP2-AS1(dist=155522) |
| Al | 7 | 73882011 | | T | | G | | 0.0137 | 7.82E-06 | -1.14 | 0.254 | intronic | GTF2IRD1 |
| Al | 9 | 107876666 | | T | | C | | 0.0585 | 8.69E-06 | -0.494 | 0.111 | intergenic | ABCA1(dist=186139),SLC44A1(dist=130228) |
| al | 2 | 34538579 | | C | | A | | 0.0105 | 8.89E-06 | 1.30 | 0.293 | intergenic | MYADML(dist=585295),NONE(dist=NONE) |
| al | 7 | 136945107 | | T | | C | | 0.239 | 9.31E-06 | 0.243 | 0.0549 | intronic | PTN |
| al | 15 | 84803344 | | A | | C | | 0.0169 | 9.31E-06 | 1.09 | 0.245 | intergenic | EFTUD1P1(dist=7991),UBE2Q2L(dist=37898) |
| cd | 6 | 74457830 | | T | | C | | 0.0175 | 1.35E-10 | -1.16 | 0.180 | intronic | CD109 |
| cd | 4 | 22306890 | | A | | T | | 0.0143 | 1.43E-07 | 1.12 | 0.213 | intergenic | KCNIP4(dist=356516),LOC100505912(dist=22100) |
| cd | 11 | 44507543 | | T | | C | | 0.435 | 1.54E-07 | 0.243 | 0.0463 | intergenic | ALX4(dist=175827),CD82(dist=79598) |
| cd | 11 | 68859870 | | G | | A | | 0.422 | 6.86E-07 | -0.288 | 0.0580 | intergenic | TPCN2(dist=1798),RP11-554A11.8(dist=54826) |
| cd | 7 | 127643968 | | C | | T | | 0.0378 | 7.71E-07 | 0.638 | 0.129 | intronic | SND1 |
| cd | 10 | 18645086 | | T | | C | | 0.162 | 8.32E-07 | 0.307 | 0.0622 | intronic | CACNB2 |
| cd | 3 | 74772076 | | C | | A | | 0.0175 | 9.24E-07 | -1.22 | 0.250 | intergenic | CNTN3(dist=201733),MIR4444-1(dist=491551) |
| cd | 7 | 110586940 | | C | | T | | 0.0173 | 9.29E-07 | 1.29 | 0.262 | intronic | IMMP2L |
| cd | 7 | 30288256 | | G | | C | | 0.0202 | 9.54E-07 | 1.06 | 0.217 | intergenic | MTURN(dist=85875),ZNRF2(dist=35667) |
| cd | 15 | 49195281 | | T | | C | | 0.0201 | 1.03E-06 | -0.789 | 0.161 | intronic | SHC4 |
| cd | 16 | 24205678 | | A | | G | | 0.0108 | 1.05E-06 | 1.73 | 0.354 | intronic | PRKCB |
| cd | 4 | 55430998 | | G | | T | | 0.451 | 1.30E-06 | -0.233 | 0.0481 | intergenic | PDGFRA(dist=266586),KIT(dist=93097) |
| cd | 15 | 28714945 | | G | | A | | 0.447 | 2.73E-06 | -0.326 | 0.0696 | intergenic | MIR4509-3(dist=43215),MIR4509-3(dist=20953) |
| cd | 10 | 7989475 | | G | | A | | 0.0852 | 3.34E-06 | 0.403 | 0.0867 | intronic | TAF3 |
| cd | 3 | 169730124 | | A | | G | | 0.181 | 3.58E-06 | 0.290 | 0.0625 | intergenic | SEC62(dist=13963),GPR160(dist=25611) |
| cd | 6 | 78769132 | | G | | A | | 0.0109 | 4.82E-06 | 1.13 | 0.248 | intergenic | MEI4(dist=134656),IRAK1BP1(dist=808129) |
| cd | 16 | 81643746 | | A | | G | | 0.0265 | 5.15E-06 | 0.666 | 0.146 | intronic | CMIP |
| cd | 16 | 13310075 | | T | | G | | 0.394 | 5.57E-06 | 0.217 | 0.0477 | intronic | SHISA9 |
| cd | 11 | 121919647 | | G | | T | | 0.0362 | 5.66E-06 | 0.606 | 0.134 | intergenic | SORL1(dist=415176),MIR100HG(dist=40164) |
| cd | 2 | 114875550 | | G | | A | | 0.0177 | 5.72E-06 | 0.849 | 0.187 | intergenic | LINC01191(dist=110663),DPP10(dist=324349) |
| cd | 13 | 61466895 | | C | | G | | 0.0187 | 6.96E-06 | 0.773 | 0.172 | intergenic | LINC00378(dist=196961),MIR3169(dist=307037) |
| cd | 7 | 3028590 | | C | | T | | 0.0350 | 7.02E-06 | 0.644 | 0.143 | intronic | CARD11 |
| cd | 6 | 151134333 | | C | | T | | 0.206 | 7.10E-06 | 0.289 | 0.0643 | intronic | PLEKHG1 |
| cd | 3 | 81678329 | | C | | G | | 0.0114 | 7.79E-06 | 1.00 | 0.224 | intronic | GBE1 |
| cd | 4 | 7940685 | | C | | T | | 0.138 | 8.53E-06 | 0.306 | 0.0688 | intronic | AFAP1 |
| cd | 5 | 174741726 | | A | | G | | 0.167 | 8.59E-06 | -0.274 | 0.0617 | intergenic | FLJ16171(dist=318992),DRD1(dist=125949) |
| cd | 14 | 82060017 | | A | | G | | 0.190 | 8.69E-06 | 0.260 | 0.0584 | intergenic | SEL1L(dist=59812),RP11-799P8.1(dist=11674) |
| cd | 5 | 57832007 | | T | | G | | 0.0163 | 8.94E-06 | -0.860 | 0.194 | intergenic | GAPT(dist=39822),LOC101928600(dist=5387) |
| cd | 4 | 57481564 | | A | | T | | 0.0408 | 8.97E-06 | 0.787 | 0.177 | intergenic | THEGL(dist=12075),HOPX(dist=32590) |
| cd | 3 | 134016577 | | A | | G | | 0.0242 | 9.06E-06 | -0.792 | 0.178 | intergenic | RYK(dist=46991),AMOTL2(dist=57610) |
| co | 4 | 54800985 | | C | | T | | 0.0116 | 7.08E-08 | 1.70 | 0.315 | intergenic | LNX1-AS2(dist=329437),RPL21P44(dist=50681) |
| co | 10 | 48934756 | | C | | G | | 0.0561 | 5.43E-07 | 0.626 | 0.125 | ncrA_intronic | BMS1P1,BMS1P5 |
| co | 12 | 87928973 | | C | | T | | 0.0125 | 9.31E-07 | -1.29 | 0.264 | intergenic | MGAT4C(dist=696292),MKRN9P(dist=247690) |
| co | 1 | 61254718 | | G | | T | | 0.0491 | 1.36E-06 | -0.696 | 0.144 | intergenic | C1orf87(dist=715276),NFIA(dist=288228) |
| co | 3 | 65841295 | | A | | G | | 0.366 | 1.55E-06 | -0.290 | 0.0604 | intronic | MAGI1 |
| co | 1 | 18513954 | | A | | C | | 0.0108 | 2.34E-06 | -1.28 | 0.271 | intronic | IGSF21 |
| co | 4 | 87117983 | | G | | T | | 0.0142 | 2.47E-06 | 1.32 | 0.281 | intronic | MAPK10 |
| co | 16 | 85371133 | | G | | C | | 0.0564 | 2.55E-06 | -0.470 | 0.100 | intergenic | MIR5093(dist=31202),GSE1(dist=273896) |
| co | 10 | 52468946 | | T | | C | | 0.459 | 2.96E-06 | -0.251 | 0.0537 | intergenic | SGMS1(dist=85209),ASAH2B(dist=30742) |
| co | 15 | 90189758 | | A | | C | | 0.0564 | 3.27E-06 | -0.474 | 0.102 | intronic | KIF7 |
| co | 1 | 234984169 | | C | | T | | 0.0181 | 4.09E-06 | -1.02 | 0.221 | intergenic | LINC01132(dist=116779),TOMM20(dist=288489) |
| co | 9 | 136311932 | | A | | G | | 0.0120 | 4.96E-06 | 1.06 | 0.232 | intronic | ADAMTS13 |
| co | 9 | 115415254 | | A | | C | | 0.0671 | 6.17E-06 | -0.413 | 0.0913 | intronic | KIAA1958 |
| co | 1 | 211129340 | | T | | C | | 0.336 | 6.63E-06 | 0.216 | 0.0480 | intronic | KCNH1 |
| co | 18 | 59117206 | | G | | T | | 0.408 | 7.00E-06 | 0.203 | 0.0453 | intergenic | MC4R(dist=1077205),CDH20(dist=40569) |
| co | 1 | 113343976 | | C | | T | | 0.0125 | 7.30E-06 | 0.993 | 0.221 | intergenic | FAM19A3(dist=74120),RP11-426L16.8(dist=18815) |
| co | 10 | 80853186 | | C | | T | | 0.0818 | 7.35E-06 | -0.386 | 0.0860 | intronic | ZMIZ1 |
| co | 3 | 124805328 | | T | | C | | 0.0187 | 7.68E-06 | 0.982 | 0.219 | intronic | SLC12A8 |
| co | 12 | 83155140 | | A | | G | | 0.0123 | 8.94E-06 | 1.22 | 0.274 | intronic | TMTC2 |
| cr | 14 | 38372295 | | C | | T | | 0.0105 | 6.33E-07 | -1.70 | 0.341 | intergenic | FOXA1(dist=307970),SSTR1(dist=304909) |
| cr | 1 | 247460727 | | A | | G | | 0.263 | 1.34E-06 | 0.258 | 0.0534 | intergenic | VN1R5(dist=40280),ZNF496(dist=2895) |
| cr | 8 | 6877545 | | A | | T | | 0.0354 | 1.42E-06 | 0.779 | 0.162 | intergenic | DEFA1(dist=1722),DEFA11P(dist=8578) |
| cr | 14 | 88275066 | | C | | T | | 0.0130 | 1.63E-06 | -1.08 | 0.225 | intergenic | LOC283585(dist=885967),GALC(dist=124292) |
| cr | 11 | 76430186 | | C | | T | | 0.454 | 1.94E-06 | 0.269 | 0.0564 | ncrA_intronic | GUCY2EP |
| cr | 21 | 25792902 | | G | | T | | 0.111 | 2.28E-06 | 0.370 | 0.0783 | intergenic | AP000469.2(dist=99212),LOC339622(dist=419962) |
| cr | 2 | 83160378 | | T | | C | | 0.149 | 2.39E-06 | -0.304 | 0.0645 | intergenic | LOC1720(dist=75485),FUNDC2P2(dist=1357428) |
| cr | 12 | 40831213 | | A | | G | | 0.0748 | 2.55E-06 | -0.416 | 0.0884 | intronic | MUC19 |
| cr | 1 | 243295612 | | C | | T | | 0.0411 | 2.56E-06 | 0.757 | 0.161 | intronic | CEP170 |
| cr | 8 | 8086541 | | T | | G | | 0.0564 | 2.59E-06 | 0.720 | 0.153 | ncrA_intronic | FAM86B3P |
| cr | 17 | 20455017 | | A | | G | | 0.0511 | 3.85E-06 | 0.672 | 0.145 | intergenic | KRT16P3(dist=47206),CDRT15L2(dist=28020) |
| cr | 2 | 104735460 | | G | | A | | 0.0554 | 5.00E-06 | 0.505 | 0.111 | intergenic | TMEM182(dist=1301322),LOC100287010(dist=259848) |
| cr | 1 | 73897116 | | T | | C | | 0.0155 | 5.42E-06 | -1.21 | 0.267 | intergenic | NEGR1(dist=1148839),LRRIQ3(dist=594586) |
| cr | 11 | 94389331 | | C | | G | | 0.0570 | 5.49E-06 | -0.468 | 0.103 | intergenic | PIWIL4(dist=34744),AMOTL1(dist=112177) |
| cr | 15 | 91114376 | | A | | G | | 0.305 | 5.52E-06 | 0.228 | 0.0502 | intronic | CRTC3 |
| cr | 2 | 111225671 |  | C |  | A |  | 0.443 | 6.08E-06 | 0.275 | 0.0608 | splicing | LIMS3(NM_033514:exon3:c.342+1G>T),LIMS3L(NM_001205288:exon3:c.342+1G>T) |
| cr | 2 | 24674550 | | A | | C | | 0.0450 | 7.38E-06 | -0.578 | 0.129 | intergenic | ITSN2(dist=91153),NCOA1(dist=132796) |
| cr | 15 | 54381684 | | A | | C | | 0.0158 | 7.73E-06 | 0.863 | 0.193 | intronic | UNC13C |
| cr | 2 | 131190102 | | G | | A | | 0.0359 | 8.06E-06 | 0.801 | 0.179 | intergenic | FAR2P2(dist=3983),CYP4F62P(dist=3133) |
| cr | 14 | 102503947 | | C | | T | | 0.0130 | 9.52E-06 | -1.15 | 0.259 | intronic | DYNC1H1 |
| cu | 1 | 98777788 | | A | | T | | 0.0111 | 9.59E-08 | -1.31 | 0.245 | intergenic | RP5-1070A16.1(dist=39574),SNX7(dist=349448) |
| cu | 2 | 146587342 | | G | | A | | 0.0414 | 1.80E-07 | 0.675 | 0.129 | intergenic | TEX41(dist=753051),PABPC1P2(dist=757283) |
| cu | 3 | 32465361 | | G | | A | | 0.0135 | 4.29E-07 | 1.02 | 0.203 | intronic | CMTM7 |
| cu | 1 | 221720239 | | C | | T | | 0.448 | 4.38E-07 | 0.217 | 0.0429 | intergenic | C1orf140(dist=210601),DUSP10(dist=154525) |
| cu | 16 | 15121147 | | G | | A | | 0.0128 | 6.38E-07 | -1.44 | 0.262 | intronic | PDXDC1 |
| cu | 16 | 10434894 | | A | | C | | 0.0113 | 6.51E-07 | -1.37 | 0.275 | intergenic | GRIN2A(dist=158283),ATF7IP2(dist=45018) |
| cu | 15 | 82881755 | | G | | A | | 0.381 | 6.98E-07 | 0.253 | 0.0510 | intergenic | RPS17L(dist=56890),ADAMTS7P1(dist=1191) |
| cu | 1 | 13777869 | | T | | A | | 0.0905 | 7.44E-07 | -0.482 | 0.0973 | intergenic | PRAMEF20(dist=30066),LRRC38(dist=23576) |
| cu | 2 | 242387786 | | A | | G | | 0.0685 | 7.52E-07 | -0.421 | 0.0850 | intronic | FARP2 |
| cu | 3 | 103311962 | | A | | G | | 0.0201 | 8.51E-07 | -1.03 | 0.208 | intergenic | ZPLD1(dist=1113277),MIR548A3(dist=591514) |
| cu | 6 | 5436160 | | A | | G | | 0.0460 | 8.93E-07 | -0.518 | 0.105 | intronic | FARS2 |
| cu | 20 | 6831233 | | C | | T | | 0.0370 | 9.54E-07 | 0.561 | 0.115 | intergenic | BMP2(dist=70323),MIR8062(dist=521022) |
| cu | 10 | 75140744 | | G | | A | | 0.0157 | 1.49E-06 | -1.25 | 0.261 | intronic | ANXA7 |
| cu | 16 | 84735483 | | A | | G | | 0.310 | 1.68E-06 | -0.221 | 0.0462 | intronic | USP10 |
| cu | 7 | 135476143 | | G | | A | | 0.0116 | 1.93E-06 | 1.35 | 0.284 | intergenic | FAM180A(dist=42549),LUZP6(dist=135360) |
| cu | 17 | 19022936 | | T | | C | | 0.301 | 1.96E-06 | 0.292 | 0.0613 | intergenic | GRAP(dist=72600),GRAPL(dist=7846) |
| cu | 20 | 52537101 | | G | | A | | 0.0121 | 1.98E-06 | -1.42 | 0.299 | intergenic | SUMO1P1(dist=44853),BCAS1(dist=22978) |
| cu | 1 | 225227512 | | G | | A | | 0.318 | 1.99E-06 | 0.242 | 0.0510 | intronic | DNAH14 |
| cu | 4 | 103331062 | | A | | G | | 0.0465 | 2.14E-06 | 0.635 | 0.134 | intergenic | SLC39A8(dist=64407),NFKB1(dist=91424) |
| cu | 4 | 174476098 | | G | | A | | 0.126 | 2.36E-06 | -0.304 | 0.0643 | intergenic | HAND2-AS1(dist=13117),FBXO8(dist=681712) |
| cu | 16 | 55905676 | | C | | T | | 0.104 | 2.62E-06 | 0.389 | 0.0829 | splicing | CES5A(NM_145024:exon4:c.279-1G>A,NM_001143685:exon4:c.279-1G>A,NM_001190158:exon5:c.366-1G>A) |
| cu | 6 | 142778663 | | C | | T | | 0.0132 | 3.36E-06 | 1.11 | 0.238 | intergenic | GPR126(dist=11260),RP11-440G9.1(dist=68929) |
| cu | 1 | 14670351 | | A | | G | | 0.0334 | 3.47E-06 | -0.676 | 0.146 | intergenic | PRDM2(dist=518777),KAZN(dist=254862) |
| cu | 16 | 28771786 | | A | | G | | 0.331 | 3.55E-06 | -0.248 | 0.0536 | intronic | NPIPB9 |
| cu | 16 | 13219035 | | C | | G | | 0.112 | 3.64E-06 | -0.315 | 0.0680 | intronic | SHISA9 |
| cu | 2 | 56251882 | | G | | A | | 0.0429 | 3.75E-06 | -0.513 | 0.111 | intergenic | MIR216B(dist=23952),CCDC85A(dist=159376) |
| cu | 14 | 97176623 | | G | | C | | 0.0145 | 3.76E-06 | -0.921 | 0.199 | intergenic | PAPOLA(dist=143170),VRK1(dist=87061) |
| cu | 22 | 26541088 | | T | | C | | 0.405 | 4.86E-06 | 0.193 | 0.0421 | intergenic | MYO18B(dist=114081),SEZ6L(dist=24352) |
| cu | 14 | 100152269 | | C | | T | | 0.0663 | 4.92E-06 | 0.411 | 0.0900 | intronic | CYP46A1 |
| cu | 10 | 132665951 | | G | | T | | 0.0158 | 5.11E-06 | -1.09 | 0.238 | intergenic | GLRX3(dist=687305),MIR378C(dist=94900) |
| cu | 22 | 45396060 | | G | | A | | 0.0286 | 5.58E-06 | -0.589 | 0.130 | intronic | PHF21B |
| cu | 15 | 93911523 | | C | | T | | 0.313 | 6.42E-06 | 0.207 | 0.0458 | intergenic | RGMA(dist=279080),MCTP2(dist=929907) |
| cu | 7 | 55658720 | | G | | A | | 0.0100 | 6.51E-06 | -1.37 | 0.305 | intergenic | VOPP1(dist=18520),FKBP9L(dist=90047) |
| cu | 14 | 89479436 | | G | | T | | 0.0291 | 6.57E-06 | -0.596 | 0.132 | intergenic | TTC8(dist=135096),FOXN3(dist=143080) |
| cu | 3 | 186577127 | | G | | A | | 0.0144 | 6.72E-06 | -1.21 | 0.269 | downstream | ADIPOQ |
| cu | 4 | 138357631 | | A | | G | | 0.134 | 7.06E-06 | -0.285 | 0.0633 | intergenic | LINC00613(dist=1522796),PCDH18(dist=82443) |
| cu | 4 | 106804941 | | C | | T | | 0.0342 | 7.43E-06 | -0.658 | 0.147 | intergenic | GSTCD(dist=36059),NPNT(dist=11656) |
| cu | 17 | 73538666 | | C | | T | | 0.0106 | 7.62E-06 | -1.09 | 0.244 | intronic | LLGL2 |
| cu | 6 | 169374497 | | G | | A | | 0.0115 | 8.02E-06 | 1.23 | 0.275 | intergenic | SMOC2(dist=305823),THBS2(dist=241378) |
| cu | 17 | 11316117 | | A | | G | | 0.301 | 8.27E-06 | 0.231 | 0.0518 | intronic | SHISA6 |
| cu | 16 | 50599680 | | C | | T | | 0.0170 | 8.68E-06 | -0.985 | 0.222 | intronic | NKD1 |
| cu | 7 | 136089950 | | A | | T | | 0.317 | 9.21E-06 | -0.208 | 0.0468 | intergenic | LUZP6(dist=427746),CHRM2(dist=463449) |
| hg | 1 | 244216257 | | C | | T | | 0.0100 | 9.23E-06 | -1.51 | 0.341 | intronic | ZBTB18 |
| hg | 3 | 177349580 | | A | | T | | 0.0140 | 1.76E-09 | -1.80 | 0.299 | ncrA_intronic | LINC00578 |
| hg | 1 | 228927279 | | T | | A | | 0.0108 | 4.14E-08 | -1.87 | 0.342 | intergenic | RHOU(dist=44863),RAB4A(dist=479530) |
| hg | 8 | 7088171 | | A | | G | | 0.152 | 3.77E-07 | 0.448 | 0.0882 | intergenic | DEFA5(dist=173912),LINC00965(dist=29970) |
| hg | 3 | 73715128 | | T | | C | | 0.0101 | 5.87E-07 | -1.24 | 0.248 | intergenic | PDZRN3(dist=41056),CNTN3(dist=596594) |
| hg | 1 | 227555822 | | C | | T | | 0.0133 | 1.96E-06 | -1.32 | 0.277 | intergenic | CDC42BPA(dist=49996),ZNF678(dist=195398) |
| hg | 10 | 89827616 | | G | | C | | 0.204 | 2.64E-06 | 0.272 | 0.0580 | intergenic | PTEN(dist=99084),RNLS(dist=206005) |
| hg | 7 | 55499145 | | C | | A | | 0.0146 | 2.87E-06 | 1.33 | 0.283 | UTR3 | LANCL2 |
| hg | 8 | 87887614 | | G | | A | | 0.0674 | 2.95E-06 | 0.466 | 0.0998 | intronic | CNBD1 |
| hg | 4 | 132653185 | | G | | C | | 0.0234 | 4.34E-06 | 0.932 | 0.203 | intergenic | NONE(dist=NONE),PCDH10(dist=1417285) |
| hg | 10 | 2193920 | | T | | C | | 0.0821 | 4.53E-06 | -0.403 | 0.0879 | intergenic | MIR6072(dist=75637),LINC00701(dist=148593) |
| hg | 14 | 55329274 | | C | | T | | 0.299 | 4.91E-06 | -0.234 | 0.0512 | intronic | GCH1 |
| hg | 4 | 64283291 | | A | | C | | 0.0222 | 4.95E-06 | 0.987 | 0.216 | intergenic | LPHN3(dist=1345123),TECRL(dist=860886) |
| hg | 12 | 132386398 | | C | | T | | 0.0249 | 5.10E-06 | 0.963 | 0.211 | intronic | ULK1 |
| hg | 22 | 49251399 | | T | | C | | 0.0221 | 6.06E-06 | -1.03 | 0.227 | intergenic | MIR4535(dist=75234),WI2-81516E3.1(dist=11183) |
| hg | 13 | 44166876 | | T | | C | | 0.0336 | 6.25E-06 | -0.573 | 0.127 | intronic | ENOX1 |
| hg | 17 | 43704790 | | C | | T | | 0.0238 | 6.25E-06 | 1.06 | 0.236 | ncrA_intronic | MGC57346 |
| hg | 17 | 50486197 | | G | | A | | 0.0280 | 6.44E-06 | 0.890 | 0.197 | intergenic | CA10(dist=248820),C17orf112(dist=576683) |
| hg | 8 | 59943898 | | T | | C | | 0.172 | 6.46E-06 | -0.279 | 0.0619 | intronic | TOX |
| hg | 7 | 10487586 | | C | | T | | 0.0141 | 6.66E-06 | -1.16 | 0.257 | intergenic | PER4(dist=812139),NDUFA4(dist=483994) |
| hg | 9 | 126858466 | | A | | T | | 0.0311 | 6.78E-06 | -0.697 | 0.155 | intergenic | LHX2(dist=63024),NEK6(dist=161419) |
| hg | 7 | 43300002 | | G | | A | | 0.0158 | 7.03E-06 | 0.922 | 0.205 | intronic | HECW1 |
| hg | 1 | 14929571 | | T | | C | | 0.146 | 7.96E-06 | -0.294 | 0.0659 | intronic | KAZN |
| hg | 4 | 154441016 | | A | | T | | 0.0110 | 9.07E-06 | 1.34 | 0.302 | intronic | KIAA0922 |
| mn | 12 | 44093038 | | A | | G | | 0.0192 | 9.59E-06 | -0.824 | 0.186 | intergenic | ADAMTS20(dist=147314),PUS7L(dist=29372) |
| mn | 1 | 220080028 | | A | | G | | 0.182 | 2.17E-14 | -0.456 | 0.0596 | ncrA_intronic | RNU5F-1 |
| mn | 4 | 103188709 | | C | | T | | 0.0380 | 5.08E-11 | -0.767 | 0.117 | exonic | SLC39A8 |
| mn | 5 | 151725452 | | C | | T | | 0.0197 | 2.44E-07 | 1.36 | 0.263 | intergenic | CTB-12O2.1(dist=75442),NMUR2(dist=45650) |
| mn | 1 | 208411806 | | T | | C | | 0.0195 | 1.01E-06 | -0.983 | 0.201 | intronic | PLXNA2 |
| mn | 3 | 85338197 | | A | | C | | 0.0128 | 1.33E-06 | -1.20 | 0.249 | intronic | CADM2 |
| mn | 9 | 122997106 | | G | | A | | 0.0118 | 1.46E-06 | -1.59 | 0.330 | intergenic | BRINP1(dist=865367),MIR147A(dist=10151) |
| mn | 11 | 127416532 | | G | | A | | 0.0167 | 1.53E-06 | -1.17 | 0.243 | intergenic | KIRREL3-AS3(dist=540579),ETS1(dist=912124) |
| mn | 18 | 56041822 | | C | | G | | 0.178 | 1.73E-06 | 0.294 | 0.0614 | intronic | NEDD4L |
| mn | 7 | 106770872 | | A | | G | | 0.0228 | 1.76E-06 | 1.00 | 0.210 | intronic | PRKAR2B |
| mn | 8 | 74840034 | | G | | A | | 0.272 | 1.82E-06 | 0.266 | 0.0558 | intergenic | UBE2W(dist=48889),TCEB1(dist=17339) |
| mn | 5 | 116971885 | | G | | A | | 0.0113 | 1.90E-06 | 1.23 | 0.258 | intergenic | LINC00992(dist=56446),LOC102467224(dist=94171) |
| mn | 10 | 61146582 | | T | | C | | 0.0128 | 2.42E-06 | 1.06 | 0.225 | intergenic | FAM13C(dist=23921),SLC16A9(dist=263940) |
| mn | 4 | 94047788 | | A | | G | | 0.103 | 2.50E-06 | -0.428 | 0.0909 | intronic | GRID2 |
| mn | 3 | 126035297 | | G | | T | | 0.368 | 2.62E-06 | 0.262 | 0.0557 | intergenic | ALDH1L1-AS2(dist=106286),KLF15(dist=26181) |
| mn | 17 | 18338397 | | A | | T | | 0.210 | 2.78E-06 | 0.373 | 0.0796 | intergenic | LOC339240(dist=9750),KRT16P1(dist=4825) |
| mn | 12 | 19870167 | | T | | C | | 0.0126 | 3.55E-06 | -1.16 | 0.251 | intergenic | AEBP2(dist=194994),RP11-664H17.1(dist=297452) |
| mn | 11 | 116111308 | | G | | A | | 0.0688 | 3.63E-06 | 0.455 | 0.0982 | intergenic | LINC00900(dist=480390),BUD13(dist=507578) |
| mn | 8 | 37485751 | | C | | A | | 0.0682 | 3.85E-06 | -0.419 | 0.0906 | intergenic | KCNU1(dist=692108),ZNF703(dist=67550) |
| mn | 12 | 1019300 | | G | | T | | 0.0284 | 5.09E-06 | -0.909 | 0.199 | UTR3 | WNK1 |
| mn | 17 | 6160874 | | T | | C | | 0.0218 | 5.09E-06 | -0.750 | 0.164 | intergenic | WSCD1(dist=133127),AIPL1(dist=166183) |
| mn | 8 | 36497380 | | A | | G | | 0.0242 | 5.26E-06 | 0.828 | 0.182 | intergenic | UNC5D(dist=845199),KCNU1(dist=144462) |
| mn | 17 | 59505389 | | G | | A | | 0.0162 | 5.51E-06 | 1.14 | 0.251 | intergenic | C17orf82(dist=14748),TBX4(dist=28418) |
| mn | 12 | 96928024 | | G | | A | | 0.0136 | 5.58E-06 | 1.40 | 0.307 | intergenic | CDK17(dist=133658),NEDD1(dist=372977) |
| mn | 13 | 21801885 | | G | | A | | 0.0302 | 5.59E-06 | 0.667 | 0.147 | intergenic | MRP63(dist=48665),MIPEPP3(dist=70379) |
| mn | 18 | 55405584 | | G | | A | | 0.156 | 5.81E-06 | -0.297 | 0.0654 | intronic | ATP8B1 |
| mn | 4 | 22678824 | | T | | G | | 0.0139 | 5.86E-06 | 1.38 | 0.304 | intergenic | GPR125(dist=161147),GBA3(dist=15713) |
| mn | 4 | 156822497 | | A | | G | | 0.432 | 5.92E-06 | 0.211 | 0.0466 | intergenic | ASIC5(dist=35072),TDO2(dist=2348) |
| mn | 10 | 105438913 | | G | | A | | 0.0111 | 5.94E-06 | 1.27 | 0.280 | intronic | SH3PXD2A |
| mn | 14 | 94812686 | | G | | T | | 0.0337 | 6.04E-06 | 0.778 | 0.172 | intergenic | SERPINA6(dist=22998),SERPINA1(dist=30398) |
| mn | 4 | 9671428 | | T | | C | | 0.0224 | 6.07E-06 | -1.05 | 0.231 | intergenic | MIR548I2(dist=113491),DRD5(dist=111830) |
| mn | 3 | 52851702 | | C | | T | | 0.0196 | 6.25E-06 | 0.798 | 0.177 | intronic | ITIH4 |
| mn | 3 | 10457857 | | T | | C | | 0.247 | 6.88E-06 | 0.238 | 0.0529 | intronic | ATP2B2 |
| mn | 9 | 139727632 | | C | | G | | 0.0174 | 7.53E-06 | -1.22 | 0.272 | intronic | RABL6 |
| mn | 10 | 60366824 | | T | | C | | 0.0131 | 7.53E-06 | -1.43 | 0.319 | intronic | BICC1 |
| mn | 4 | 179431345 | | T | | G | | 0.0134 | 8.13E-06 | 1.11 | 0.249 | intergenic | LINC01098(dist=519441),NONE(dist=NONE) |
| mn | 14 | 28146992 | | A | | G | | 0.0772 | 8.34E-06 | -0.402 | 0.0902 | intergenic | LINC00645(dist=38150),FOXG1(dist=1089286) |
| mn | 8 | 69756024 | | C | | T | | 0.0111 | 9.38E-06 | -1.03 | 0.233 | intergenic | C8orf34(dist=24766),RP11-600K15.1(dist=68014) |
| mn | 4 | 182590856 | | C | | T | | 0.0212 | 9.56E-06 | -0.731 | 0.165 | intergenic | LINC00290(dist=510554),MGC45800(dist=469303) |
| mo | 4 | 59249282 | | A | | G | | 0.0874 | 9.81E-06 | -0.507 | 0.115 | intergenic | IGFBP7-AS1(dist=1177817),NONE(dist=NONE) |
| mo | 3 | 24824730 | | C | | T | | 0.0110 | 2.42E-07 | 1.32 | 0.255 | intergenic | MIR4792(dist=261804),RARB(dist=645024) |
| mo | 10 | 16973499 | | G | | A | | 0.0109 | 3.38E-07 | -1.67 | 0.328 | intronic | CUBN |
| mo | 8 | 95855720 | | A | | G | | 0.0219 | 4.24E-07 | -1.11 | 0.219 | intronic | INTS8 |
| mo | 10 | 69644473 | | T | | G | | 0.0195 | 4.40E-07 | -1.12 | 0.222 | UTR5 | SIRT1 |
| mo | 6 | 20415005 | | C | | T | | 0.133 | 1.23E-06 | -0.338 | 0.0697 | intronic | E2F3 |
| mo | 14 | 42034549 | | G | | A | | 0.0128 | 2.20E-06 | 1.56 | 0.329 | intergenic | LOC644919(dist=424298),LRFN5(dist=42215) |
| mo | 16 | 79120562 | | G | | A | | 0.0821 | 2.26E-06 | -0.413 | 0.0874 | intronic | WWOX |
| mo | 8 | 144152551 | | G | | T | | 0.0215 | 2.77E-06 | -0.984 | 0.210 | intergenic | C8orf31(dist=16831),LY6H(dist=86780) |
| mo | 4 | 160131148 | | A | | T | | 0.0256 | 3.31E-06 | -0.731 | 0.157 | intergenic | MIR3688-1(dist=81102),RAPGEF2(dist=57850) |
| mo | 4 | 106048563 | | G | | A | | 0.112 | 4.25E-06 | -0.344 | 0.0748 | intergenic | CXXC4(dist=632505),TET2(dist=18469) |
| mo | 7 | 47023974 | | A | | T | | 0.284 | 6.33E-06 | -0.238 | 0.0527 | intergenic | IGFBP3(dist=1063103),TNS3(dist=290778) |
| mo | 12 | 122163276 | | G | | A | | 0.359 | 6.38E-06 | 0.212 | 0.0469 | intronic | TMEM120B |
| mo | 1 | 24489358 | | T | | C | | 0.0621 | 6.44E-06 | -0.613 | 0.136 | intronic | IFNLR1 |
| mo | 1 | 12859260 | | T | | C | | 0.171 | 7.34E-06 | 0.268 | 0.0597 | intergenic | PRAMEF1(dist=2483),PRAMEF11(dist=25208) |
| mo | 1 | 66013531 | | G | | A | | 0.0617 | 8.11E-06 | -0.520 | 0.117 | intronic | LEPR |
| mo | 7 | 19855434 | | C | | T | | 0.221 | 8.38E-06 | -0.308 | 0.0692 | intergenic | TMEM196(dist=43030),AC005062.2(dist=103170) |
| mo | 3 | 86753198 | | A | | G | | 0.0156 | 9.45E-06 | -1.17 | 0.264 | intergenic | RNU6-69P(dist=262837),VGLL3(dist=233925) |
| ni | 6 | 44524666 | | G | | C | | 0.312 | 9.51E-06 | 0.219 | 0.0494 | intergenic | CDC5L(dist=106505),SUPT3H(dist=269801) |
| ni | 19 | 21582973 | | G | | A | | 0.0303 | 1.17E-07 | -0.814 | 0.154 | intronic | ZNF493 |
| ni | 1 | 155371135 | | C | | T | | 0.0184 | 4.79E-07 | -1.29 | 0.256 | intronic | ASH1L |
| ni | 8 | 10929021 | | T | | C | | 0.169 | 9.53E-07 | -0.370 | 0.0755 | intronic | XKR6 |
| ni | 18 | 23078929 | | C | | G | | 0.103 | 1.93E-06 | 0.365 | 0.0767 | intergenic | ZNF521(dist=146715),SS18(dist=517288) |
| ni | 6 | 123641670 | | A | | G | | 0.0150 | 1.98E-06 | -1.23 | 0.258 | intronic | TRDN |
| ni | 19 | 681193 | | C | | T | | 0.201 | 2.12E-06 | 0.293 | 0.0618 | intronic | FSTL3 |
| ni | 20 | 5424021 | | T | | C | | 0.0108 | 2.79E-06 | 1.50 | 0.320 | ncrA_intronic | LINC00658 |
| ni | 15 | 73598548 | | T | | C | | 0.475 | 2.80E-06 | 0.219 | 0.0468 | intergenic | NEO1(dist=1001),HCN4(dist=13652) |
| ni | 16 | 10725409 | | G | | A | | 0.0152 | 2.85E-06 | 1.06 | 0.227 | intronic | TEKT5 |
| ni | 4 | 3798508 | | G | | T | | 0.229 | 3.14E-06 | -0.267 | 0.0574 | intergenic | ADRA2C(dist=28255),FAM86EP(dist=145161) |
| ni | 18 | 51115162 | | C | | T | | 0.0106 | 3.15E-06 | 1.51 | 0.324 | intergenic | DCC(dist=52889),MBD2(dist=562809) |
| ni | 16 | 8303285 | | A | | T | | 0.0344 | 3.17E-06 | 0.733 | 0.157 | intergenic | RBFOX1(dist=539945),TMEM114(dist=316217) |
| ni | 14 | 56367569 | | C | | G | | 0.0139 | 4.25E-06 | 0.996 | 0.217 | intergenic | LINC00520(dist=104177),PELI2(dist=217524) |
| ni | 1 | 5158445 | | A | | G | | 0.0481 | 4.59E-06 | -0.529 | 0.115 | intergenic | AJAP1(dist=314594),MIR4417(dist=465686) |
| ni | 13 | 37151895 | | C | | T | | 0.0758 | 5.04E-06 | -0.398 | 0.0873 | intergenic | CCNA1(dist=134876),SERTM1(dist=96154) |
| ni | 6 | 31080586 | | A | | G | | 0.0935 | 5.06E-06 | -0.376 | 0.0823 | upstream | C6orf15 |
| ni | 13 | 79837542 | | A | | G | | 0.0106 | 5.38E-06 | 1.23 | 0.269 | intergenic | LINC00331(dist=423357),RBM26(dist=55461) |
| ni | 8 | 141895555 | | G | | A | | 0.0140 | 5.40E-06 | 1.08 | 0.237 | intronic | PTK2 |
| ni | 10 | 6840536 | | T | | A | | 0.0258 | 6.26E-06 | 0.925 | 0.205 | ncrA_intronic | LINC00707 |
| ni | 15 | 61495458 | | G | | A | | 0.388 | 6.58E-06 | 0.206 | 0.0457 | intronic | RORA |
| ni | 20 | 56887156 | | C | | T | | 0.0700 | 6.96E-06 | -0.410 | 0.0912 | intronic | RAB22A |
| ni | 5 | 122899301 | | C | | T | | 0.232 | 7.50E-06 | -0.246 | 0.0550 | intronic | CSNK1G3 |
| ni | 14 | 61614856 | | C | | T | | 0.0396 | 7.52E-06 | 0.629 | 0.140 | intergenic | SLC38A6(dist=64405),TMEM30B(dist=129233) |
| ni | 1 | 59088629 | | A | | C | | 0.0154 | 8.10E-06 | 1.15 | 0.259 | intergenic | TACSTD2(dist=45463),MYSM1(dist=31782) |
| ni | 10 | 68014687 | | C | | A | | 0.0358 | 8.19E-06 | 0.567 | 0.127 | intronic | CTNNA3 |
| ni | 10 | 104290462 | | C | | T | | 0.313 | 8.28E-06 | 0.221 | 0.0495 | intronic | SUFU |
| ni | 1 | 212290542 | | G | | A | | 0.453 | 9.04E-06 | -0.218 | 0.0491 | intergenic | DTL(dist=12194),PPP2R5A(dist=168337) |
| ni | 8 | 6826043 | | A | | T | | 0.254 | 9.04E-06 | -0.245 | 0.0552 | ncrA_intronic | DEFA10P |
| pb | 10 | 130656497 | | C | | T | | 0.144 | 9.28E-06 | -0.288 | 0.0650 | intergenic | MKI67(dist=732029),MGMT(dist=608957) |
| pb | 1 | 249059465 | | T | | C | | 0.0470 | 2.29E-07 | -0.649 | 0.126 | intergenic | LYPD8(dist=156314),SH3BP5L(dist=45186) |
| pb | 3 | 114107799 | | G | | T | | 0.488 | 2.45E-07 | 0.241 | 0.0467 | ncrA_exonic | ZBTB20-AS1 |
| pb | 6 | 109031577 | | T | | C | | 0.146 | 2.89E-07 | 0.325 | 0.0633 | intergenic | FOXO3(dist=25606),LINC00222(dist=41280) |
| pb | 16 | 83930574 | | A | | G | | 0.242 | 3.05E-07 | -0.270 | 0.0527 | intergenic | HSBP1(dist=83967),MLYCD(dist=2156) |
| pb | 19 | 40039093 | | A | | G | | 0.0300 | 4.44E-07 | -0.716 | 0.142 | intergenic | EID2(dist=8255),LGALS13(dist=54076) |
| pb | 14 | 50807206 | | G | | C | | 0.0147 | 8.26E-07 | 1.33 | 0.270 | intronic | CDKL1 |
| pb | 14 | 85184779 | | A | | G | | 0.0524 | 9.80E-07 | -0.511 | 0.104 | intergenic | NONE(dist=NONE),LINC00911(dist=675444) |
| pb | 12 | 55411303 | | G | | C | | 0.0154 | 1.24E-06 | 1.16 | 0.239 | intergenic | TESPA1(dist=32773),NEUROD4(dist=2426) |
| pb | 11 | 7301162 | | C | | T | | 0.497 | 1.47E-06 | 0.218 | 0.0453 | intronic | SYT9 |
| pb | 12 | 123730522 | | T | | C | | 0.0175 | 1.73E-06 | 0.953 | 0.199 | intronic | C12orf65 |
| pb | 13 | 88298813 | | C | | A | | 0.0114 | 1.92E-06 | 1.29 | 0.271 | ncrA_intronic | MIR4500HG |
| pb | 4 | 148303054 | | T | | C | | 0.0152 | 2.13E-06 | 1.07 | 0.225 | intergenic | TTC29(dist=436020),EDNRA(dist=99015) |
| pb | 14 | 76311122 | | A | | T | | 0.0801 | 2.50E-06 | 0.397 | 0.0844 | intronic | TTLL5 |
| pb | 1 | 25439449 | | A | | G | | 0.0624 | 4.73E-06 | -0.448 | 0.0979 | intergenic | RUNX3(dist=147948),SYF2(dist=109318) |
| pb | 1 | 185263310 | | G | | C | | 0.124 | 4.92E-06 | 0.332 | 0.0726 | intergenic | SWT1(dist=2397),IVNS1ABP(dist=2212) |
| pb | 9 | 78543660 | | A | | G | | 0.173 | 5.61E-06 | -0.280 | 0.0616 | intronic | PCSK5 |
| pb | 5 | 81955213 | | C | | T | | 0.0548 | 5.63E-06 | 0.440 | 0.0968 | intergenic | ATP6AP1L(dist=341066),MIR3977(dist=180761) |
| pb | 9 | 2229313 | | G | | C | | 0.0646 | 5.97E-06 | 0.420 | 0.0927 | intergenic | SMARCA2(dist=35690),VLDLR-AS1(dist=306342) |
| pb | 5 | 27572907 | | A | | G | | 0.0665 | 6.07E-06 | 0.453 | 0.100 | intergenic | LINC01021(dist=76399),LSP1P3(dist=1354070) |
| pb | 14 | 45563049 | | C | | T | | 0.0482 | 7.05E-06 | -0.517 | 0.115 | intronic | PRPF39 |
| pb | 3 | 4920165 | | C | | A | | 0.0378 | 7.21E-06 | 0.587 | 0.131 | intergenic | ITPR1(dist=30641),BHLHE40-AS1(dist=19749) |
| pb | 4 | 23646922 | | G | | T | | 0.233 | 7.59E-06 | 0.252 | 0.0563 | intergenic | MIR548AJ2(dist=182196),PPARGC1A(dist=146722) |
| pb | 16 | 25092316 | | G | | A | | 0.290 | 7.89E-06 | 0.227 | 0.0509 | intergenic | LOC554206(dist=48218),LCMT1(dist=30731) |
| pb | 15 | 44049734 | | C | | T | | 0.0152 | 8.58E-06 | 1.19 | 0.269 | intronic | PDIA3 |
| pb | 15 | 53430936 | | C | | G | | 0.119 | 8.59E-06 | 0.421 | 0.0946 | intergenic | ONECUT1(dist=348727),WDR72(dist=375002) |
| pb | 13 | 112531114 | | G | | A | | 0.0207 | 9.32E-06 | -0.774 | 0.175 | intergenic | TEX29(dist=534520),SOX1(dist=190799) |
| pb | 12 | 123187273 | | G | | A | | 0.0114 | 9.44E-06 | 1.05 | 0.236 | exonic | HCAR2 |
| zn | 16 | 11926943 | | T | | C | | 0.0165 | 9.84E-06 | 1.12 | 0.253 | intergenic | BCAR4(dist=4254),RSL1D1(dist=1112) |
| zn | 11 | 92932229 | | C | | T | | 0.474 | 2.17E-07 | -0.231 | 0.0446 | intergenic | SLC36A4(dist=1088),CCDC67(dist=131654) |
| zn | 8 | 42906157 | | T | | G | | 0.0919 | 6.03E-07 | 0.391 | 0.0783 | intergenic | HOOK3(dist=20475),FNTA(dist=5285) |
| zn | 1 | 188883764 | | C | | G | | 0.0144 | 7.53E-07 | -1.43 | 0.289 | intergenic | PLA2G4A(dist=1925651),BRINP3(dist=1183033) |
| zn | 2 | 66586934 | | C | | T | | 0.364 | 7.90E-07 | -0.236 | 0.0478 | intergenic | MIR4778(dist=1474),MEIS1-AS3(dist=63541) |
| zn | 17 | 53322324 | | A | | G | | 0.416 | 8.64E-07 | -0.224 | 0.0456 | intergenic | STXBP4(dist=80875),HLF(dist=19997) |
| zn | 9 | 100305082 | | A | | G | | 0.386 | 1.11E-06 | -0.225 | 0.0463 | intronic | TMOD1 |
| zn | 6 | 110914481 | | G | | A | | 0.439 | 1.40E-06 | -0.215 | 0.0445 | intergenic | SLC22A16(dist=116637),CDK19(dist=16700) |
| zn | 8 | 96524590 | | T | | C | | 0.233 | 3.11E-06 | 0.262 | 0.0562 | ncrA_intronic | LOC100616530 |
| zn | 5 | 88462456 | | G | | A | | 0.0334 | 3.54E-06 | 0.740 | 0.160 | intergenic | MEF2C-AS1(dist=134263),MIR3660(dist=849982) |
| zn | 3 | 145561990 | | T | | C | | 0.167 | 4.25E-06 | 0.273 | 0.0594 | intergenic | C3orf58(dist=1850780),PLOD2(dist=225238) |
| zn | 14 | 20401656 | | A | | T | | 0.381 | 4.33E-06 | 0.262 | 0.0569 | intergenic | OR4K5(dist=11919),OR4K1(dist=2111) |
| zn | 6 | 55488643 | | A | | G | | 0.0335 | 4.39E-06 | -0.671 | 0.146 | intergenic | HMGCLL1(dist=44631),BMP5(dist=131595) |
| zn | 2 | 31724549 | | G | | A | | 0.489 | 5.66E-06 | 0.218 | 0.0479 | intergenic | XDH(dist=86938),SRD5A2(dist=25107) |
| zn | 6 | 9021556 | | G | | A | | 0.184 | 5.73E-06 | -0.289 | 0.0636 | intergenic | RP11-314C16.1(dist=235878),TFAP2A(dist=1375360) |
| zn | 1 | 6207217 | | T | | C | | 0.391 | 5.82E-06 | 0.205 | 0.0453 | intronic | CHD5 |
| zn | 8 | 86260295 | | A | | T | | 0.390 | 5.94E-06 | -0.215 | 0.0475 | intronic | CA1 |
| zn | 5 | 147811995 | | G | | A | | 0.0157 | 6.21E-06 | -1.08 | 0.240 | intronic | FBXO38 |
| zn | 3 | 106092346 | | G | | A | | 0.460 | 6.77E-06 | -0.208 | 0.0461 | intergenic | CBLB(dist=504459),LINC00882(dist=736291) |
| zn | 3 | 6045748 | | G | | A | | 0.0105 | 7.83E-06 | -1.36 | 0.305 | intergenic | MIR4790(dist=753808),AC069277.1(dist=628297) |
| zn | 8 | 81588870 | | G | | T | | 0.0108 | 8.05E-06 | -1.05 | 0.234 | intronic | ZNF704 |
| zn | 7 | 1320239 | | G | | A | | 0.403 | 8.33E-06 | 0.252 | 0.0566 | intergenic | UNCX(dist=43626),MICALL2(dist=153756) |
| zn | 5 | 56535889 | | T | | A | | 0.0180 | 8.53E-06 | -0.837 | 0.188 | intronic | GPBP1 |
| zn | 7 | 18157048 | | G | | A | | 0.0211 | 8.81E-06 | 0.806 | 0.181 | intronic | HDAC9 |
| zn | 14 | 26364940 | | C | | T | | 0.454 | 8.95E-06 | 0.207 | 0.0465 | intergenic | STXBP6(dist=845845),NOVA1(dist=550149) |
| zn | 6 | 69358032 | | G | | A | | 0.0391 | 9.65E-06 | 0.553 | 0.125 | intronic | BAI3 |
| zn | 1 | 229278334 | | C | | T | | 0.185 | 9.70E-06 | -0.261 | 0.0590 | intergenic | RHOU(dist=395918),RAB4A(dist=128475) |

Figure 1 – Local association plots for manganese


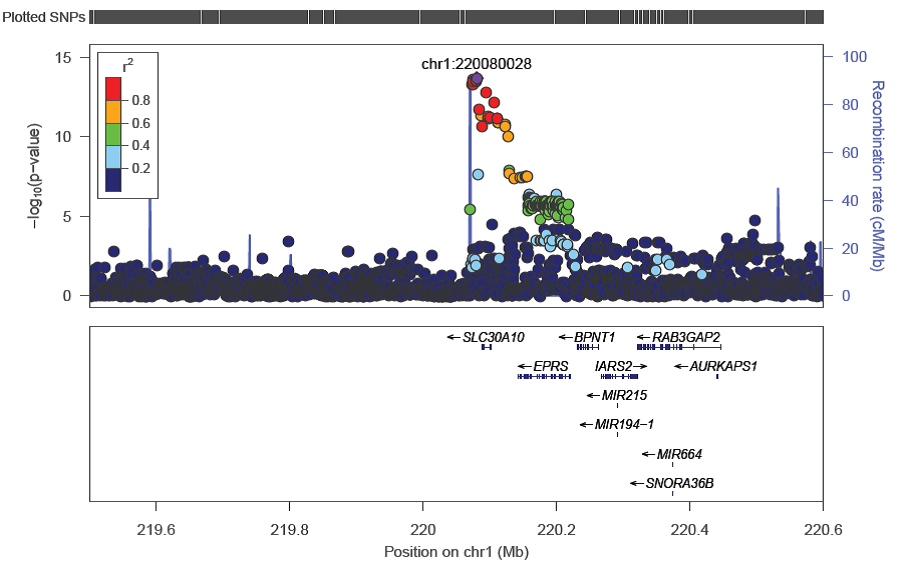


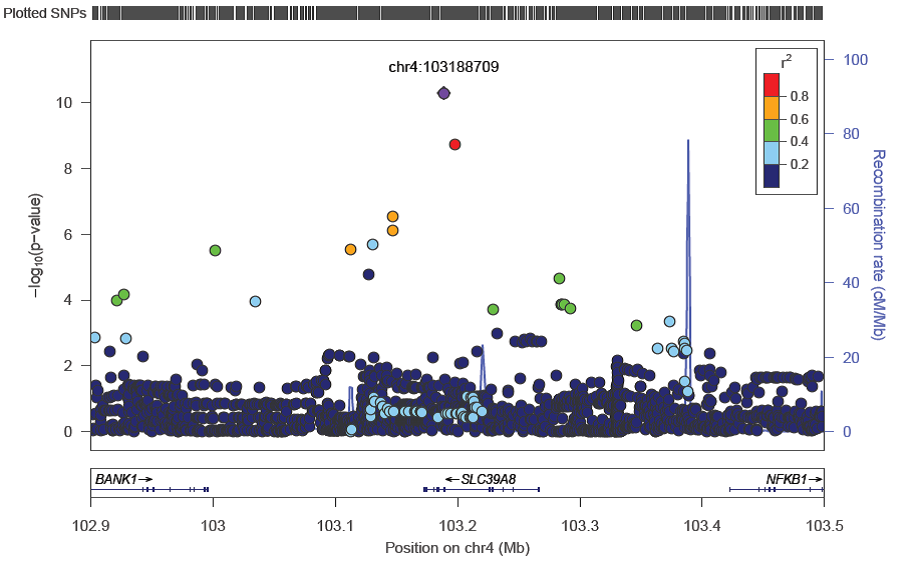


Each point represents a SNP plotted with their p-value (on a -log10 scale) as a function of genomic position (NCBI Build 37). In each panel, the lead SNP is represented by the purple diamond. The colour coding of all other SNPs (circles) indicates LD with the lead SNP (estimated by CEU r2 from the 1000 Genomes Project March 2012 release): red r2≥0.8; gold 0.6≤r2<0.8; green 0.4≤r2<0.6; cyan 0.2≤r2<0.4; blue r2<0.2; grey r2 unknown. Recombination rates are estimated from the International HapMap Project and gene annotations are taken from the University of California Santa Cruz genome browser. Each point corresponds to a SNP passing QC, plotted according to genomic position on the x-axis and the strength of association (-log10 p-value) on the y-axis. The red line indicates the genome wide significance threshold (5x10^-8^), while the blue line indicates a threshold of 10^-05^

Figure 2 - Local association plot for cadmium

Each point represents a SNP plotted with their p-value (on a -log10 scale) as a function of genomic position (NCBI Build 37). In each panel, the lead SNP is represented by the purple diamond. The colour coding of all other SNPs (circles) indicates LD with the lead SNP (estimated by CEU r2 from the 1000 Genomes Project March 2012 release): red r2≥0.8; gold 0.6≤r2<0.8; green 0.4≤r2<0.6; cyan 0.2≤r2<0.4; blue r2<0.2; grey r2 unknown. Recombination rates are estimated from the International HapMap Project and gene annotations are taken from the University of California Santa Cruz genome browser.


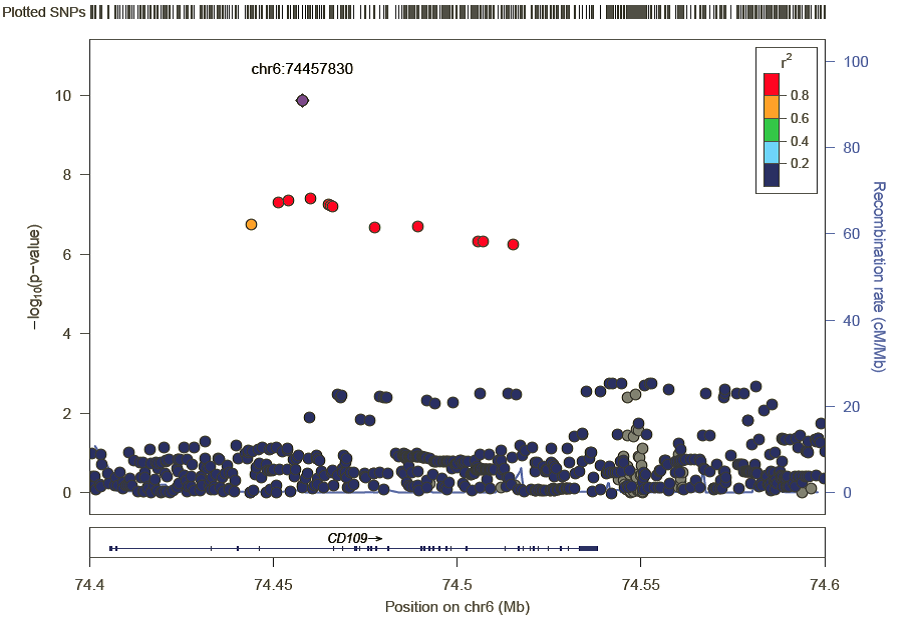


Figure 3 – Local association plot for mercury

Each point represents a SNP plotted with their p-value (on a -log10 scale) as a function of genomic position (NCBI Build 37). In each panel, the lead SNP is represented by the purple diamond. The colour coding of all other SNPs (circles) indicates LD with the lead SNP (estimated by CEU r2 from the 1000 Genomes Project March 2012 release): red r2≥0.8; gold 0.6≤r2<0.8; green 0.4≤r2<0.6; cyan 0.2≤r2<0.4; blue r2<0.2; grey r2 unknown. Recombination rates are estimated from the International HapMap Project and gene annotations are taken from the University of California Santa Cruz genome browser


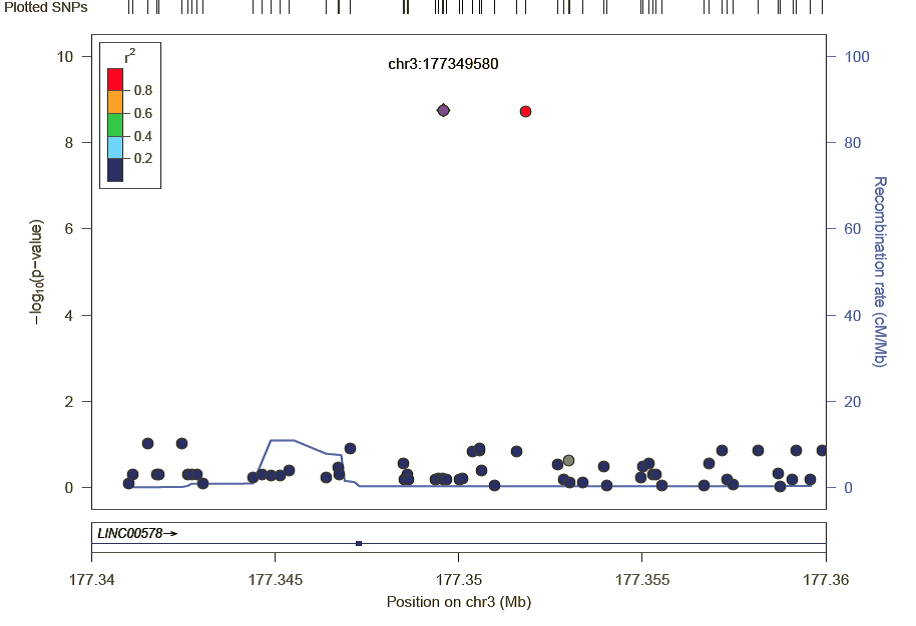

Supplement: Supplementary Data [file supp_ddv190_ddv190supp.docx]
